# Deciphering the Genetic Architecture of Color Variation in Whole Grain Rice by Genome-Wide Association

**DOI:** 10.3390/plants12040927

**Published:** 2023-02-17

**Authors:** Wenjun Wang, Xianjin Qiu, Ziqi Wang, Tianyi Xie, Wenqiang Sun, Jianlong Xu, Fan Zhang, Sibin Yu

**Affiliations:** 1 National Key Laboratory of Crop Genetic Improvement, Hubei Hongshan Laboratory, Huazhong Agricultural University, Wuhan 430070, China; 2 College of Agriculture, Yangtze University, Jingzhou 434025, China; 3 Institute of Crop Science, Chinese Academy of Agricultural Sciences, Beijing 100081, China

**Keywords:** genome-wide association, grain color, whole grain, pigmented rice, metabolite, rice

## Abstract

Whole grain rice is recommended in a natural healthy diet because of its high nutritional and healthful benefits compared to polished or white rice. The whole grain contains the pericarp with many assorted colors (such as brown, red, and black) associated with taste and commercial quality. The color attributes of whole grain or brown rice are usually undesirable and need to be improved. To decipher the genetic basis of color variation in the whole grain rice, we conducted a genome-wide association analysis of three parameters of grain colors (brightness, redness, and yellowness) in a panel of 682 rice accessions. Twenty-six loci were identified for the color parameters, implying that grain color is under polygenic control. Among them, some major-effect loci were co-localized with the previously identified genes such as *Rc* and *Rd*. To eliminate the possible mask of *Rc* on other loci influencing grain color, we performed the association analysis in a subset of the panel that excluded the pigmented (red and black) rice. Eighteen loci or SNPs were detected to be associated with grain color in the subpopulation, many of which were not reported before. Two significant peak SNP regions on chromosomes 1 and 9 were validated using near-isogenic lines. Based on differential expression analysis of annotated genes within the SNP regions and metabolic analysis of pooled extreme samples, we found at least three annotated genes as potential candidates involved in the flavonoid metabolic pathway related to pericarp color. These results provide insights into the genetic basis of rice grain color and facilitate genomic breeding to improve appearance and commercial quality of whole grain rice.

## 1. Introduction

Rice (*Oryza sativa* L.) is a major cereal crop and provides the main source of energy intake for over half of the global population. Milled or polished rice is usually consumed, because of its translucent and good taste in major rice-eating countries. However, the milling process of rice alters the nutrient composition and sensory attributes such as texture and color [1]. The polished rice obtained by removing the bran layer leads to a lack of essential vitamins, minerals, and other functional nutrient compounds [2]. Whole grain rice is the version of the unpolished grain (traditionally called brown rice) that contains the pericarp, aleurone, and germ. It is recommended in a naturally healthy diet and has gradually been accepted in developing countries, due to a valuable source of various nutritional and bioactive compounds that positively impact disease prevention [3,4,5].

The appearance quality of whole grain rice or brown rice greatly affects the market competitiveness. In particular, the color and texture of whole grain rice change the sensory perception and affect consumer acceptance. For example, Asian consumers preferred rice with a white appearance [6]. The grain color ranges from white to red, purple, and black in the pericarp because of some extant gathering of various flavonoids and phenolic metabolites [7,8]. Most rice varieties have a light brown or white pericarp, usually treated as white or non-pigmented rice; there is also pigmented rice, such as red and black rice, which accumulate proanthocyanidin and anthocyanin in the pericarp, respectively [8].

It is well-documented that many structural and regulatory genes associated with flavonoid biosynthesis confer grain colors in some pigmented cereals. Some major-effect genes for grain pigmentation have already been identified involving anthocyanin and proanthocyanidin biosynthesis [9]. For example, several key genes in the biosynthesis of anthocyanin, such as chalcone synthase (CHS), chalcone isomerase (CHI), dihydroflavonol 4-reductase (DFR), anthocyanin synthase (ANS), and UDP-glycosyltransferase (UGT), are associated with grain colors in cereal crops [3,10,11,12,13,14]. In rice, pericarp color is controlled by *OsMYB3/Kala3*, *OsB2/Ra2/Kala4*, *Rc*, and *Rd/Kala1* [15,16,17]. The R2R3-MYB gene *OsMYB3/Kala3* participates in the biosynthesis of anthocyanin [18]. The basic helix-loop-helix (bHLH) transcription factor *OsB2/Kala4* controls anthocyanin production in the pericarp, producing black grains [19]. Meanwhile, *Rc* encoding a bHLH protein confers proanthocyanidin accumulation in the pericarp. The loss-of-function mutant *Rc* causes the change from red to white grains [20]. *Rc* interacts with *Rd*, producing red grains; *Rd* encoding a form of DFR enhances proanthocyanidin content in the pericarp. *Rc* generates brown grains in the absence of *Rd*, whereas *Rd* alone has no color phenotype [21,22].

In addition to the above-mentioned genes inferring the genetic variation in grain pigmentation, there are numerous quantitative trait loci or single nucleotide polymorphisms (SNPs) associated with color variation in rice grains [23,24,25,26,27]. These results indicate a polygenic inheritance of grain color. In addition, transcriptomic profiling and metabolomic analysis have identified certain structural and regulatory genes influencing the flavonoid biosynthesis pathway [28,29,30,31]. However, the genes and their metabolic products that explain the genetic variation in non-pigmented or white rice remain elusive.

To analyze the genetic basis of grain color, we carried out genome-wide association analysis (GWAS) of three parameters, brightness or lightness (L*), redness (a*), and yellowness (b*), in a panel of rice germplasms including pigmented and white rice. With the aim to explore the genetic variation in whole grain rice, we integrated near-isogenic lines with transcription profile and metabolic analysis to pinpoint candidate genes with significant SNPs associated with grain color. In addition to the major gene *Rc*, some candidate genes were identified for grain color variation. The results provide valuable information for the rice industry and the development of nutritional rice varieties to meet multiple needs.

## 2. Results

### 2.1. Large Variation in Rice Grain Color

**Figure 1:**
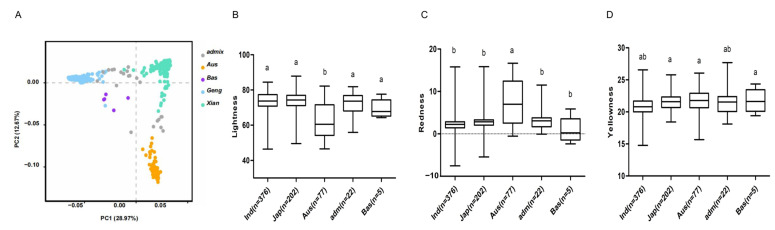
Principal component analysis (PC) of rice accessions based on single nucleotide polymorphisms indicates the population structure with five major groups. (**A**) PC analysis shows two main components; (**B**–**D**) brightness, redness, and yellowness of grain color. *Ind*, *Indica* (*Xian*); *Jap*, *Japonica* (*Geng*); *Aus*, *Aus* group; *Adm*, admixture; *Bas*, *Basmati* group; *n*, number of accessions. The different lowercase letters above the boxplot indicate significant differences among the five groups by LSD test.

**Figure 2:**
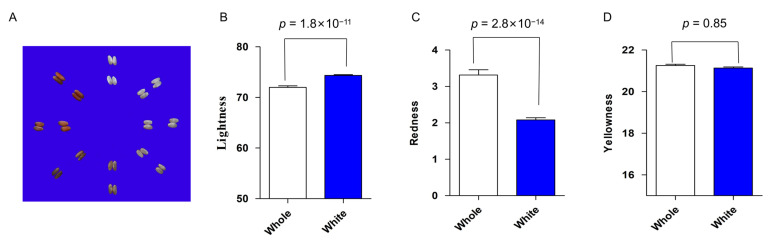
Genetic variation in grain color in rice germplasm. (**A**) Representative grain colors in the whole population, (**B**) brightness, (**C**) redness, (**D**) yellowness; *p*-values are given by Student’s *t*-test.

A panel of 682 genome-sequenced rice accessions, including 597 white rice and 85 red or pigmented varieties, was divided into five groups (*indica*, *japonica*, *Aus*, *Bas*, and *admix*). Among them, 376 accessions belong to the *indica* group, and 202 varieties are classed into the *japonica* group. A principal component (PC) analysis of the population reveals that the first two PCs dominated the population structure and explained approximately 41.6% of the genetic variation (Figure 1A). In the population exists a large variance in grain color as reflected by the three parameters, ranging from 46.4 to 87.8 for brightness, from −7.5 to 16.7 for redness, and from 14.8 to 27.7 for yellowness (Figure 1B–D). In addition, brightness has a significant correlation with redness (r = −0.76, *p* < 0.0001), and yellowness is neither correlated with brightness nor redness. We observed significant differences in the two parameters brightness and redness among the five groups. The *Aus* group shows the significantly lowest mean value of brightness, while its mean value of redness is the highest of the five groups (Figure 1B,C). Notably, the whole population was also visually divided into white rice and red (or pigmented) rice, of which the white reveals a significantly higher mean value of brightness (74.2), a lower value of redness (2.2) than the whole with the pigmented (red) rice, but no significance in yellowness between the whole population and the white subpopulation (Figure 2A–D).

### 2.2. Genome-Wide Association Analysis of Grain Color

GWAS was performed for the three parameters of grain color in the whole population consisting of white and pigmented rice and in the subpopulation without the pigmented rice. 

**Figure 3:**
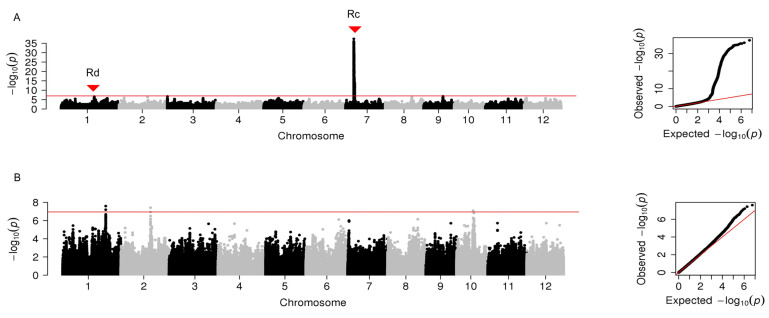
Manhattan plots of genome-wide association analysis for grain brightness in 12 chromosomes and quantile-quantile plots of *p*-values conducted in the whole population (**A**) and the subpopulation that excluded pigmented rice (**B**). The horizontal red line represents the significance threshold of *p*-value 2.23 × 10^−6^. Two cloned genes are indicated with solid triangles.

In the whole population, three significant peak SNPs were identified for brightness (L*) and distributed on chromosomes 1, 7, and 9, respectively (Figure 3A, Table S1). Among them, the peak SNP on chromosome 7 explained the largest phenotypic variation (39.3%); it overlapped with the previously reported gene *Rc*. The peak SNP on chromosome 1, nearby the *Rd* gene, explained phenotypic variation (0.9%).

In the subpopulation, three peak SNPs were significantly associated with brightness and located on chromosomes 1, 2, and 10, respectively (Figure 3B, Table S2). Notably, *Rc* and *Rd* were undetected in the subpopulation.

**Figure 4:**
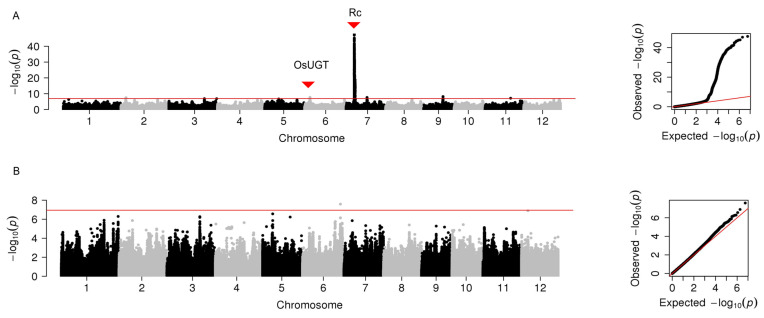
Manhattan plots of genome-wide association analysis for grain redness in 12 chromosomes and quantile-quantile plots of *p*-values conducted in the whole population (**A**) and the subpopulation that excluded pigmented rice (**B**). The horizontal red line represents the significance threshold of *p*-value 2.23 × 10^−6^. Two previously identified genes for flavonoid biosynthesis are indicated with solid triangles.

For the redness (a*), six peak SNPs were identified on chromosomes 2, 3, 6, 7, and 9, respectively. Among them, the peak SNPs on chromosome 6 overlapped with *OsUGT* and explained phenotypic variation (22.9%). The peak SNPs on chromosome 7 localized with *Rc* had the most considerable contribution to the phenotypic variation (42.2%) in the whole population (Figure 4A, Table S1). However, no locus with consecutive SNPs was identified for redness in the subpopulation that excluded pigmented rice (Figure 4B).

**Figure 5:**
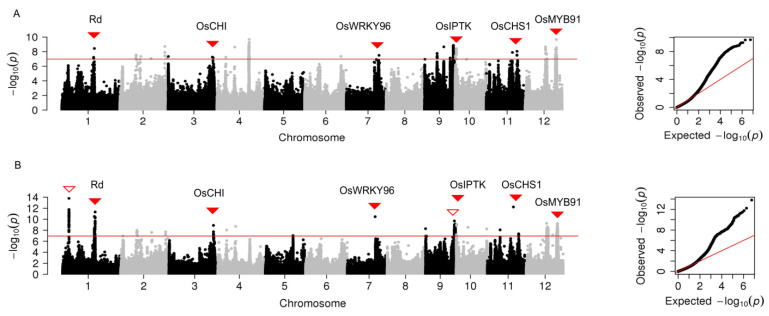
Manhattan plots of genome-wide association analysis for grain yellowness in 12 chromosomes and quantile-quantile plots of *p*-values conducted in the whole population (**A**) and the subpopulation that excluded pigmented rice (**B**). The horizontal red line represents the significance threshold of *p*-value 2.23 × 10^−6^. Several previously identified genes are indicated with solid triangles, and two novel candidate genes are highlighted with hollow triangles.

For the yellowness (b*), 17 significant peak SNPs were identified in the whole population (Table S1). Fifteen significant SNPs were detected in the subpopulation in which the pigmented accessions were removed (Table S2). Ten were found commonly in both populations (Tables S1 and S2). Among them, six peak regions were co-localized with the genes related to secondary metabolites, such as *Os01g42460* (*Rd*), *Os03g60509* (*OsCHI*), *Os07g40570* (*OsWRKY96*), *Os10g01480* (*OsIPTK*), and *Os11g32650* (*OsCHS1*) for flavonoid metabolites, and *Os12g38400* (*OsMYB91*) for phenolic metabolites (Figure 5). These genes involved in the synthesis of phenolamines and flavonoids may cause a pigment accumulation in the pericarp [3,30]. Intriguingly, four significant SNP regions were detected differently in either the whole population or the subpopulation. For example, one lead SNP (rs4708052) on chromosome 1 with the highest *p*-value (1.57 × 10^−14^) was identified in the subpopulation and explained approximately 3.5% of the yellowness variance (Figure 5B, Table S2). Another peak SNP (rs24028124) on chromosome 4 with a higher *p*-value was identified only in the whole population and explained approximately 4.5% of the yellowness variance (Figure 5A, Tables S1 and S2). These results indicate that some loci involved in grain yellowness assayed by yellowness may be independent of the previously reported genes for pigment accumulation.

### 2.3. Validation of the Effect of Lead SNP Regions on Grain Yellowness

**Figure 6:**
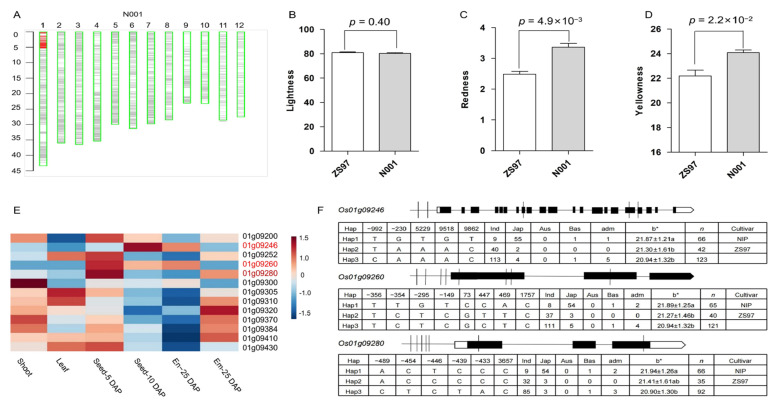
Validation of the effect of the peak region on grain color and candidate gene analysis of rs4708052 on chromosome 1. (**A**) Graphic genotype of NIL (N001) showing a single introduced segment containing the region of interest covering the candidate genes. (**B**–**D**) Three parameters of grain color of ZS97 and NIL; *p*-values are given by Student’s *t*-test. (**E**) Expression profile of candidate genes in various tissues including seedling shoot, leaf, and young seeds, embryo (em), endosperm (en) at 5, 10, or 25 days after pollination (DAP). (**F**) Schematic candidate genes with sequence variations indicated and significant differences in the mean values of grain color (column b*) between main haplotypes (Hap); *n*, number of accessions; different letters appended to the mean ± SD indicate significance at *p* < 0.05.

**Figure 7:**
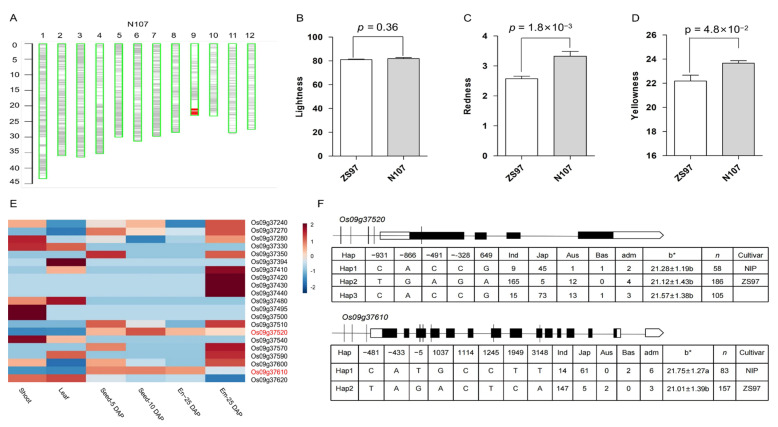
Validation of the effect of peak region on grain color and candidate gene analysis of rs21614928 on chromosome 9. (**A**) Graphic genotype of NIL (N107) showing single introduced segment containing the particular region with candidate genes. (**B**–**D**) Three parameters of grain color of ZS97 and NIL; *p*-values are given by Student’s *t*-test. (**E**) Expression profile of candidate genes in various tissues including seedling shoot, leaf, and young seeds, embryo (em), endosperm (en) at 5, 10, or 25 days after pollination (DAP). (**F**) Schematic candidate genes with sequence variations indicated and differences in the mean values of grain color (column b*) between main haplotypes (Hap); *n*, number of accessions; different letters appended to the mean ± SD indicate a significance at *p* < 0.05.

To verify the effects of the peak SNPs (rs4708052 on chromosome 1 and rs21614928 on chromosome 9) identified for grain yellowness in the subpopulation, we used a backcrossing scheme to generate near-isogenic lines (NILs) that contain only a single segment encompassing the SNPs of interest in the same background of ZS97. Two obtained NILs (N001 and N107) exhibited an apparent difference in grain color as reflected by redness and yellowness (Figure 6A and Figure 7A). The redness and yellowness values were significantly higher in the NILs than the control (ZS97), while no difference was observed in the brightness value against ZS97 (Figure 6B–D and Figure 7B–D), indicating that two peak SNP regions both influence grain yellowness.

### 2.4. Expression Analysis of Candidate Genes in Two Significant Regions

To identify potential candidate genes for the peak SNPs, we used the China Rice Data Center database to search annotated genes in a 200 kb region around the target SNPs (https://ricedata.cn/gene/, accessed on 1 November 2022). The genomic region (4.61–4.81 Mb) on chromosome 1 surrounding the peak SNP (rs4708052) contains 14 annotated genes. To narrow into the candidates, the expression profile across various tissues was analyzed (http://rice.uga.edu/cgi-bin/gbrowse/rice/, accessed on 1 December 2022). The results exhibited that *Os01g09246*, *Os01g09260*, and *Os01g09280* were predominantly expressed in young seeds at 5 and 10 days after pollination (DAP) and in the 25 DAP endosperm (Figure 6E). Further analysis of three major haplotypes (*n* > 34) of the genes based on their sequence variation (http://ricevarmap.ncpgr.cn/, accessed on 1 December 2022) revealed significant differences in yellowness between these haplotypes. Hap1 of *Os01g09246* and *Os01g09260* dominated in *japonica* and exhibited significantly higher yellowness values than the other two haplotypes, which dominated in *indica* (Figure 6F), whereas the gene *Os01g09280* had no significant difference between Hap1 and Hap2. Therefore, the former two genes (*Os01g09246* and *Os01g09260*) specifically expressed in seeds may be the most likely candidates affecting flavonoid synthesis that cause differences in pericarp color.

Similar analyses were performed for the peak SNP region (21.51–21.71 Mb) on chromosome 9. This region contains 34 annotated genes. The expression profile revealed that the two genes, *Os09g37520* and *Os09g37610*, were highly expressed in 5 DAP and 10 DAP young seeds (Figure 7E). *Os09g37520* has three major haplotypes in the whole population, but there is no significant difference in grain yellowness among the three haplotypes (Figure 7F). This gene encodes a DUF-containing protein, which contains a bZIP domain that promotes proanthocyanidin synthesis [31]. *Os09g37610* has two major haplotypes in the population, of which Hap1 is significantly higher yellowness compared to another haplotype (Figure 7F). The gene encodes a multidrug and toxin extrusion protein, which may be associated with anthocyanin transport [32].

### 2.5. Metabolite Analysis of Pooled Samples with Different Grain Colors

**Figure 8:**
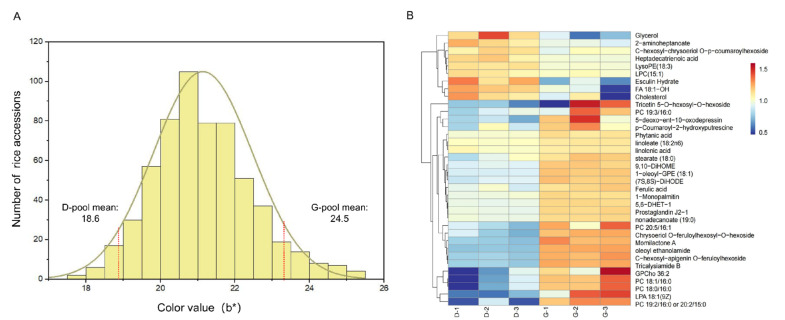
Metabolite analysis of two extreme samples pooled by yellowness. (**A**) Distribution of yellowness values in the subpopulation without pigmented rice, showing two pools with extremely low or high mean yellowness values. (**B**) Heatmap of differently expressed metabolites for the extreme pools. Three repeats for each pool (D and G pool) were conducted for metabolite analyses.

To determine whether some metabolites influence rice grain color, we selected 40 accessions with extremely higher or lower yellowness values from the white subpopulation to construct two extreme pools, each containing 20 samples for metabolite analysis (Figure 8A). The extremely high pool had the yellowness values ranging from 23.7 to 25.5, while the low pool had the yellowness values ranging from 17.7 to 18.9. The metabolic analysis of the two pools using liquid chromatography-mass spectrometry revealed 36 differentially expressed metabolites (Figure 8B). These metabolites were enriched on lipids (75.0%), flavonoids (11.1%), and terpenoids (8.3%). Most (28/36) of the differentially expressed metabolites were up-regulated in the high yellowness pool. 

Intriguingly, some key secondary metabolites such as chrysoeriol-O-feruloylhexoside, C-hexosyl-apigenin-O-feruloylhexoside, p-Coumaroyl-2-hydroxyputrescine, and ferulic acid were up-regulated by one-fold or more in the high yellowness pool compared to the low pool. In line with these data, the peak SNP region harboring *Os09g37520* and *Os09g37610* overlapped with the previously reported locus for C-pentosyl-chrysoeriol O-feruloylhexoside accumulation [30].

## 3. Discussion

Whole grain rice, particularly pigmented (red, purple, and black) rice, is becoming increasingly popular due to its high nutritional value and potential health benefits compared with milled or polished rice [3,5]. Most whole grain rice is white or non-pigmented rice. Even so, it is still rich in nutrients, such as protein, fiber, oils, minerals, and functional metabolites, and has a wide variance in grain color that significantly influence consumer acceptance. However, the genetic basis of grain color variation remains elusive. In the present study, 26 peak SNPs or loci were identified for grain colors in 682 rice accessions using a GWAS approach, indicating that rice grain color is a complex phenotype under polygenic control. Compared with the GWAS results from rice grain color in the whole population and the non-pigmented population (Tables S1 and S2), we found that grain brightness and grain redness assayed by the parameters L* and a* are controlled mainly by *Rc*, which regulates proanthocyanidin synthesis [20]. Grain yellowness assayed by b* is largely affected by minor genes, each explaining phenotypic variation of less than 5% (Figure 5, Table S2). Moreover, GWAS revealed ten significant peak SNPs or loci identified in common for yellowness in the populations no matter whether pigmented rice contained them or not. These results suggest that the expression of the minor genes for yellowness may be independent of the *Rc* regulatory pathway. *Rc* was reported as a domesticated or selected gene. The mutant *rc* was fixed in approximately 97% of white rice [20,21]. Therefore, it is comprehensible that only a few loci identified for redness in the white rice, as a small part of the redness variation was retained. In contrast, the yellowness of rice grains was neither selected for nor received any attention during rice domestication, which led to an unexpected large variance in yellowness in rice. Consistent with this case, several SNPs are identified associated with yellowness in rice germplasms. 

In the present study, genomic tools including GWAS, transcription profile, and metabolite analysis were applied to identify significant loci and candidate genes for grain color. The combined analyses largely enhance the precision of SNP identification and narrow down candidate genes directly. In this context, numerous loci with candidate genes were identified in the population of pigmented and white rice, several of them co-localizing with the previously reported genes involved in the anthocyanin or proanthocyanidin synthesis pathway, such as *Rc*, *OsCHI*, *OsMYB91*, and *OsWRKY96* [3,30,33,34]. Meanwhile, many were not reported before (Table S2). In particular, using the GWAS and NIL validation, we verified the effects of two novel peak SNPs or genomic regions on chromosomes 1 and 9 for grain color. We could nominate three potential candidate genes of these loci for grain yellowness variation through gene expression profiles, haplotype analysis, and pooled metabolite analysis. These results will help in further transgenic experiments to functionally characterize the gene(s) responsible for the variation in grain color. In addition, we found significant differences in the contents of 36 metabolic components between the two groups pooled on extreme yellowness values, most of which are flavonoids and lipids. The higher flavonoids or lipids may contribute to strong grain yellowness reflected by the higher value of the parameter b*. Previous studies revealed that the content of flavonoids was significantly correlated with the color of rice grain [35], and phenolic metabolites and flavonoids in colored rice were more than twice that of white rice [36]. Taken together, our results suggest that a wide variance of particular metabolic components exists in the pericarp or bran layers, leading to the change from white and light-colored to brown in non-pigmented rice.

Currently, as most customers prefer white rice, the cultivated high-yielding rice varieties generally have white pericarps. To meet the challenge of increasing demand for nutritional quality, it has become a priority in breeding programs to develop whole grain rice varieties with beneficial nutrients, good palatability, and favorable pericarp color [37]. Our findings provide some important insights into the genetic basis of rice grain color and the identification and manipulation of novel genes for appropriate color values of brightness, redness, and yellowness. In this context, the pyramiding of genes increased brightness and decreased yellowness, which may be an option designed to develop desirable white whole grain rice. Further exploration of natural germplasms with desired metabolites and creating molecular markers of target genes will facilitate employing genomic breeding approaches to develop whole grain rice varieties with high nutritious and commercial qualities.

## 4. Materials and Methods

### 4.1. Plant Materials

A panel of 682 rice accessions from 3K RGP was used for GWAS (Wang et al. 2018). Based on their genomic variation, the population was divided into five subgroups, *Indica* or *Xian* (376), *Japonica* or *Geng* (202), *Aus* (77), *Admix* (22), and *Bas* (5). Near-isogenic lines (NILs) that carry a particular single genomic segment of interest were constructed previously using a marker-assisted selection backcross scheme, in which the recurrent recipient was *indica* variety Zhenshan 97 (ZS97) and the donor was *japonica* variety Nipponbare (NIP). Briefly, ZS97 was crossed with NIP, and the F_1_ was backcrossed with ZS97 four times to the BC_4_F_1_ generation. At BC_4_F_2_, the NILs carrying a particular introduced segment in the similar ZS97 background were selected using an Infinium RICE6K array containing 5102 SNPs [38]. The plant materials were cultivated in the experimental field at Wuhan (30.4° N, 114.2° E). 

### 4.2. Measurement of Grain Colors

Seeds for rice accession were fresh-harvested and dried naturally, then stored in the dark at 4 °C until use. About 20 g of healthy seeds were dehulled with a rice huller (JLG-II, Institute of Grain Storage, Chengdu, China) to produce brown rice. Grain color was measured with a color difference meter (CR400, Konica Minolta, Japan). System color correction was standardized using the black and white plate provided by the CR400 instrument, with five repeated measurements for each sample. Color parameters were expressed as L*, a*, and b* values. L* is defined as the brightness or lightness index, indicating black and white, 0 is black, 100 is white, and between 0–100 is gray; a* indicates the range from red to green, and the positive value is red and the negative value is green; b* represents the range from blue to yellow, and the positive value is yellow and negative value is blue [23,25]. 

### 4.3. Genome-Wide Association Analysis

Genome-wide association analysis was performed as described in the previous study by using the 32 M SNP dataset. Briefly, the 32 M SNP dataset was downloaded from 3K RGP in the RFGB 2.0 dataset [39]. SNPs were filtered using the criteria of having less than 20% of missing data and minor allele frequency (MAF) >0.05. A total of 75,329 independent SNPs were obtained, and ADMIXTURE and GCTA software were used to analyze the population structure and principle component in the panel of rice accessions [40]. To detect trait–SNP associations for grain color, 448,430 high-quality SNPs were used in SVS software package v8.4.0 with a mixed linear model [41]. R-package (https://cran.r-project.org/package=qqman, accessed on 1 November 2022) was used to produce Manhattan and quantile-quantile plots. The threshold to declare the significant association was set as 2.23 × 10^−6^.

### 4.4. Expression Profile Analysis

Expression data of candidate genes were downloaded from the Rice Genome Annotation Project database (http://rice.uga.edu/cgi-bin/gbrowse/rice/, accessed on 10 November 2022) for analysis. The tissues include the seedling shoot, leaf, and young seeds, the embryo (em), and endosperm (en) at 5, 10, or 25 days after pollination (DAP). Data were log_10_-transformed for statistical analysis to improve normality. A heatmap for various tissue data was performed by using a heatmap package in MetaboAnalyst (https://www.metaboanalyst.ca/, accessed on 15 November 2022).

### 4.5. Metabolite Determination

Metabolite extraction was performed according to the method described previously [42]. Briefly, approximately 0.1 g of brown rice powder was extracted overnight at 4 °C with 1.0 mL of 70% aqueous methanol. After centrifuging at 12,000 rpm for 10 min, the supernatant was filtered using a syringe-facilitated 13 mm diameter nylon filter with a pore size of 0.22 μm (SCAA-104, Shanghai, China). The filtrate was dried under nitrogen gas for approximately 4 h at room temperature, and then dissolved in 200 μL of methanol. A liquid chromatography-mass spectrometry (LC-MS) system (LCMS-8060, Kyoto, Japan) was used for the relative quantification of metabolites. Metabolite data were log_2-_transformed for statistical analysis to improve normality. Student’s *t*-test was used to determine differentially present metabolites between the extreme pools at *p* < 0.05 with absolute log_2_ FC (fold change) ≥1. Heatmap for differentially present metabolites was performed in OmicStudio tools (https://www.omicstudio.cn/tool, accessed on 10 November 2022).

## 5. Conclusions

This study found large variations in three parameters of grain color in a panel of rice germplasms. Grain brightness and redness are mainly controlled by *Rc*, while grain yellowness is affected by numerous minor-effect genes. Six loci are co-localized or overlapped with the genes related to flavonoid metabolites. Eight loci are newly identified to be associated with grain color in the non-pigmented rice, of which three candidate genes, *Os01g09246*, *Os01g09260*, and *Os09g37610*, are mined for grain yellowness by genome-wide association analysis combined with transcription profile and pooled metabolic analysis. These results shed light on the genetic basis of grain color and facilitate exploring favorable genes and developing whole grain rice varieties with high nutritious and desirable commercial qualities through molecular breeding strategies.

## Data Availability

The datasets supporting the conclusions of this article are included within the article (and its Additional Files).

## Supplementary Materials

The following supporting information can be downloaded at: https://www.mdpi.com/article/10.3390/plants12040927/s1. Table S1: Significant SNPs associated with three parameters of rice grains in the whole population identified by genome-wide association study. Table S2: Significant SNPs associated with three parameters of rice grains in the subpopulation identified by genome-wide association study.
